# Supplementary material for: Quality Evaluation of the Oil of Camellia spp
Source: Foods. 2022 Jul 26;11(15):2221. doi: 10.3390/foods11152221 (PMC9368027; doi:10.3390/foods11152221)
Supplement: Supplementary file 1 [file foods-11-02221-s001.zip › foods-1818153-supplementary.pdf]

Attached Figure

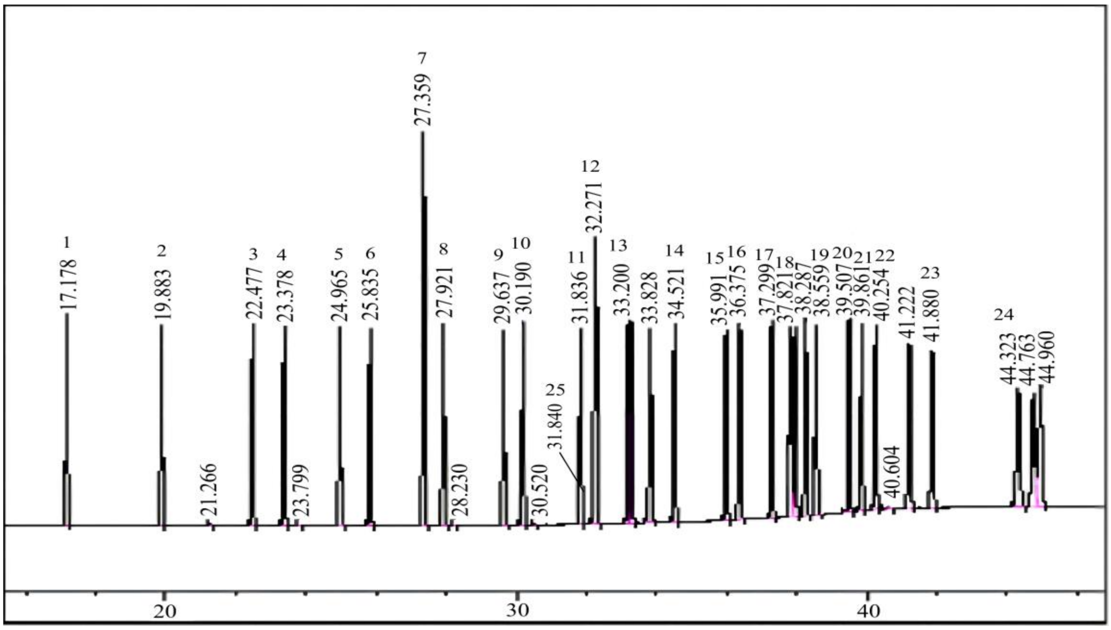

Figure S1. Standard product gas chromatography diagram. Note: 1—lauric acid, 2—tridecanoic acid, 3—myristic acid, 4—tetradecenic acid, 5—pentadecoic acid, 6—pentadecenic acid, 7—palmitic acid, 8—palmitoleic acid, 9—daturic acid, 10—heptadecenoic acid, 11—stearic acid, 12—oleic acid, 13—linoleic acid, 14—linolenic acid, 15—arachidic acid, 16—peanut monoenic acid, 17—peanut dienic acid, 18—eicosatrienoic acid, 19—arachidonic acid, 20—peanut pentenic acid, 21—docosanoic acid, 22—sinapic acid, 23—tricosanic acid, 24—lignoceric acid, 25—erucylacetic acid.

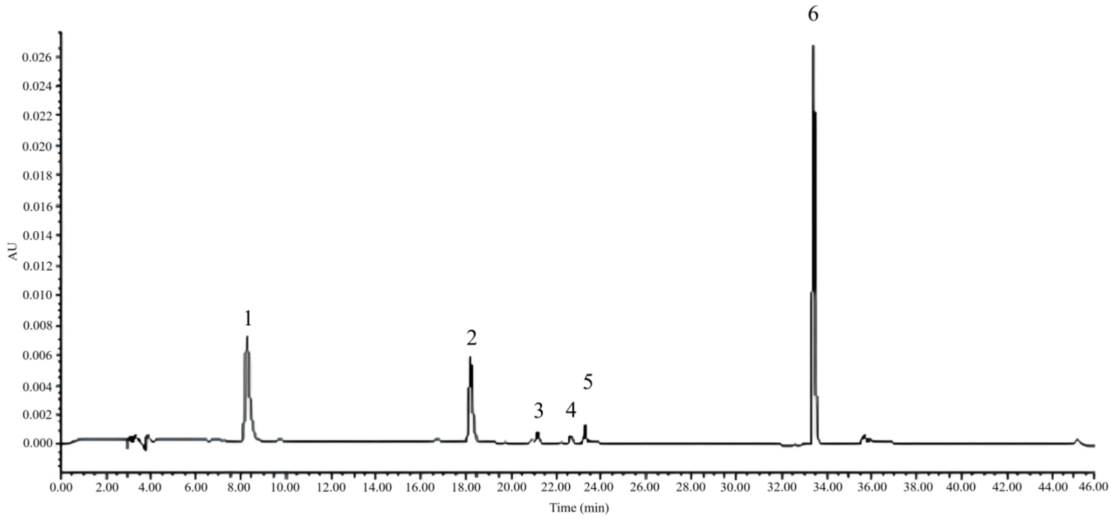

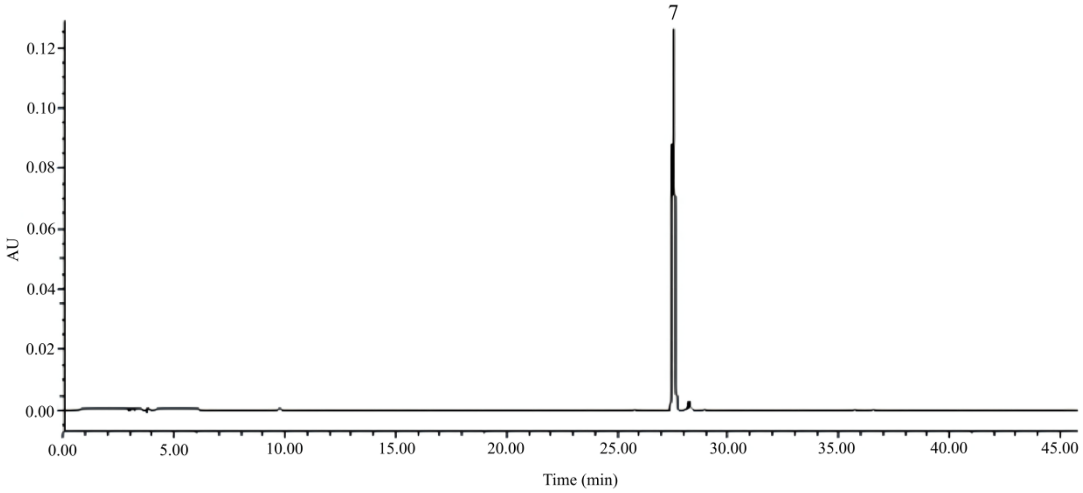

Figure S2. Standard product HPLC chromatogram map. Note: 1—3,4-dihydroxyphenylacetic acid, 2—epicatechin, 3—tea saponin A, 4—tea saponin B, 5—rutin, 6—quercetin, 7—camellianin A.

Attached table

Table S1

Standard product GC data.

Peak standard number	Retention time（min）	Peak area	Area percentage（%）	Substance
1	17.178	119.8	0.040	C12:0（lauric acid）
2	19.882	119.4	0.040	C13:0（tridecanoic acid）
3	22.477	123.4	0.042	C14:0（myristic acid）
4	23.378	120.7	0.041	C14:1 (tetradecenic acid)
5	24.965	124.4	0.042	C15:0（pentadecoic acid）
6	25.835	121.4	0.041	C15:1（pentadecenic acid）
7	27.359	253.0	0.085	C16:0（palmitic acid）
8	27.921	124.4	0.042	C16:1（palmitoleic acid）
9	29.637	127.2	0.043	C17:0（daturic acid）
10	30.191	126.1	0.043	C17:1（heptadecenoic acid）
11	31.863	129.5	0.044	C18:0（stearic acid)
12	32.271	259.6	0.088	C18:1（oleic acid）
13	33.200	131.2	0.044	C18:2（linoleic acid）
14	34.521	127.7	0.043	C18:3（linolenic acid）
15	35.991	130.7	0.044	C20:0（arachidic acid）
16	36.375	130.5	0.044	C20:1（peanut monoenic acid）
17	37.229	130.8	0.044	C20:2 (peanut dienic acid）
18	37.821	130.7	0.044	C20:3 (eicosatrienoic acid)
19	38.559	130.4	0.044	C20:4（arachidonic acid）
20	39.507	128.7	0.044	C20:5（peanut pentenic acid）
21	39.861	132.0	0.045	C22:0（docosanoic acid）
22	40.254	132.7	0.047	C22:1（sinapic acid）
23	41.880	133.0	0.045	C23:0（tricosanic acid）
24	44.323	133.5	0.045	C24:0（lignoceric acid）
25	31.840	23.8	0.973	C24:1（erucylacetic acid）

Table S2

Standard product linear equations.

Standard name	linear equation	correlation coefficent（R^2）
3,4-dihydroxyphenylacetic acid	y = 0.8724 x + 12,773.8481	1.0000
catechin	y = 1.3367 x + 10,024.2704	0.9999
tea saponin A	y = 1.3586 x + 1,788.6250	0.9987
tea saponin B	y = 1.3216 x + 802.3287	0.9999
Camellianin A	y = 1.3586 x + 1,788.6250	0.9996
rutin	y = 0.0999 x + 1,381.3163	0.9998
quercetin	y = 0.4261 x + 13096.1356	0.9997
gallic acid	y=7.7093x	0.9983
rutin	y=11.451x	0.9946
Trolox (DPPH)	y=-0.0005x+0.7644	0.9975
Trolox (ABTS)	y=-0.0007x+0.6306	0.9432
$FeSO_4$	y=0.0002x+0.0032	0.9947

Table S3

Variance contribution of quality of 40 *Camellia* spp. samples.

major constituent	eigenvalue	The contribution of variance（%）	Cumulative variance contribution rate（%）
squalene	4.852	32.345	32.345
α- tocopherol	3.043	20.285	52.631
β-sitosterol	1.5	10.002	62.633
β-amyrin	1.143	7.622	70.255
3,4-dihydroxyphenylacetic acid	1.051	7.008	77.262
epicatechin	0.77	5.133	82.395
Total saponin	0.657	4.38	86.775
rutin	0.563	3.755	90.53
Camellianin A	0.482	3.21	93.74
quercetin	0.348	2.321	96.061
Total phenol	0.196	1.308	97.369
Total flavonoids	0.174	1.159	98.528
ABTS	0.133	0.885	99.413
DPPH	0.078	0.52	99.933
FRAP	0.01	0.067	100

Table S4

Load matrix of the main component of *Camellia* spp.

	1	2	3	4	5
squalene	0.467	0.686	-0.136	0.285	0.467
α- tocopherol	0.572	0.550	-0.364	0.249	0.572
β-sitosterol	0.102	0.802	-0.241	0.119	0.102

β-amyrin	0.302	0.709	-0.047	-0.137	0.302
3,4-dihydroxyphenylacetic acid	0.395	-0.443	0.105	0.185	0.395
epicatechin	0.107	-0.353	0.225	0.015	0.107
Total saponin	0.723	-0.351	0.033	0.379	0.723
rutin	-0.647	0.311	0.089	0.162	-0.647
Camellianin A	0.556	0.144	-0.106	-0.670	0.556
quercetin	-0.463	0.401	0.375	-0.395	-0.463
Total phenol	-0.293	0.315	0.746	0.316	-0.293
Total flavonoids	0.661	0.234	0.578	0.023	0.661
ABTS	0.945	-0.248	0.025	0.034	0.945
DPPH	0.841	-0.258	0.053	-0.186	0.841
FRAP	0.670	0.345	0.416	-0.147	0.670
